# Two-stage study designs combining genome-wide association studies, tag single-nucleotide polymorphisms, and exome sequencing: accuracy of genetic effect estimates

**DOI:** 10.1186/1753-6561-5-S9-S64

**Published:** 2011-11-29

**Authors:** Laura L Faye, Shelley B Bull

**Affiliations:** 1 Dalla Lana School of Public Health, University of Toronto, Health Sciences Building, 155 College Street, Toronto, ON M5T 3M7, Canada; 2 Samuel Lunenfeld Research Institute of Mount Sinai Hospital, 60 Murray Street, Box 18, Toronto, ON M5T 3L9, Canada

## Abstract

Genome-wide association studies (GWAS) test for disease-trait associations and estimate effect sizes at tag single-nucleotide polymorphisms (SNPs), which imperfectly capture variation at causal SNPs. Sequencing studies can examine potential causal SNPs directly; however, sequencing the whole genome or exome can be prohibitively expensive. Costs can be limited by using a GWAS to detect the associated region(s) at tag SNPs followed by targeted sequencing to identify and estimate the effect size of the causal variant. Genetic effect estimates obtained from association studies can be inflated because of a form of selection bias known as the winner’s curse. Conversely, estimates at tag SNPs can be attenuated compared to the causal SNP because of incomplete linkage disequilibrium. These two effects oppose each other. Analysis of rare SNPs further complicates our understanding of the winner’s curse because rare SNPs are difficult to tag and analysis can involve collapsing over multiple rare variants. In two-stage analysis of Genetic Analysis Workshop 17 simulated data sets, we find that selection at the tag SNP produces upward bias in the estimate of effect at the causal SNP, even when the tag and causal SNPs are not well correlated. The bias similarly carries through to effect estimates for rare variant summary measures. Replication studies designed with sample sizes computed using biased estimates will be under-powered to detect a disease-causing variant. Accounting for bias in the original study is critical to avoid discarding disease-associated SNPs at follow up.

## Background

Selection bias in genetic association studies arises when the same sample is used for both gene discovery and effect estimation. Under the low power that is common in a genome-wide association study (GWAS), selection causes upward bias in the magnitude of genetic effect estimates because the effect size is estimated only when the test statistic exceeds the threshold for significance. This phenomenon is also known as the winner’s curse, and its effect on linkage analyses and on case-control association was demonstrated by Goring et al. [1] and Garner [2], respectively. In a two-stage design, the correlation between the tag single-nucleotide polymorphism (SNP) used for selection and one or more causal variants adds complexities to the understanding of the winner’s curse.

Linkage disequilibrium causes the test statistics and effect size estimates at the tag and causal SNPs to be correlated. Linkage disequilibrium can be quantified by *r*^2^ or other measures, but in this case we focus on the Pearson correlation coefficient *r* as an estimate of correlation *ρ*. Low correlation between the tag SNP and causal SNPs decreases the power to detect the effect at the tag SNP, which induces upward selection bias in both the tag and causal SNP estimates. On the other hand, as correlation decreases, the tag effect attenuates and selection at the tag SNP exerts less influence on the estimate at the causal SNP [1-3]. The balance between these two trends determines the degree of bias in the estimates.

Sequencing studies can uncover rare SNPs, for which conventional tests, such as single-SNP linear or logistic regression, are not powerful. Recently, methods to test for and estimate the effect size of multiple rare variants have been proposed. Several investigators have proposed a region-specific regression method that collapses genotypes at all rare variants in a specified region [4,5]. Within the region, *n_i_* is the number of rare SNPs genotyped for individual *i*, *r_i_* is the number of rare SNPs at which individual *i* has a rare allele, and the independent variable is *r_i_*/*n_i_*. Morris and Zeggini define the regression parameter *λ* as the genetic effect size for an individual carrying the rare allele at each of these *n_i_* rare SNPs [4].

In a two-stage design the relationship between the tag SNP and a rare causal SNP is complicated by the minor allele frequency (MAF) and the contribution of multiple causal SNPs to *r_i_*/*n_i_*. The correlation coefficient *r* between two SNPs has an upper bound that depends on the difference between the MAFs. A tag SNP with MAF > 5% will capture little of the variation at a rare SNP with MAF < 0.1%, especially in a small sample. A tag SNP with a low MAF tends to do better at capturing variation at a rare causal SNP. When multiple rare SNPs contribute to the genetic score for an individual, a useful tag SNP would be correlated with multiple SNPs.

In this paper, we study the consequences of a two-stage design for estimating the genetic effect at both the GWAS and sequencing stages. Using the Genetic Analysis Workshop 17 (GAW17) unrelated mini-exome data set [6] and the corresponding tag SNP genotypes drawn from the publicly available HapMap data set [7], we estimate the magnitude of the winner’s curse and the attenuation resulting from incomplete correlation between the tag SNP and causal SNPs. We demonstrate that selection bias occurs in both stages, even when the tag SNP is poorly correlated with the rare or the common causal SNP(s).

## Methods

We examine three different two-stage scenarios described below in which first a genetic effect is detected at a tag SNP in a GWAS, then the gene is sequenced to find the true causal SNP(s), and finally the genetic effect is estimated at the true causal SNP(s). We compare the distribution of genetic effect estimates over varying correlations between the tag SNP and the causal SNP and over varying MAFs at both SNPs. The examination of the design matrix for the additive model shows that the relevant quantity, γTC, for comparing the tag SNP and causal SNP effects is:(1)

where *p_C_* is the causal SNP MAF, *p_T_* is the tag SNP MAF, and *ρ_TC_* is the correlation between the tag SNP and causal SNPs. Without loss of generality, we use *ρ_TC_* to refer to both the correlation parameter and the Pearson correlation coefficient in the finite sample.

For analysis, we classify the GAW17 data into the following subpopulations: CEPH (European-descent Utah residents), Chinese, Japanese, Tuscan, Luhya, Yoruba, Europeans (CEPH + Tuscan), Asians (Chinese + Japanese), and Africans (Luhya + Yoruba). We define a common SNP as one with MAF > 5% and a rare SNP as one with MAF < 5% within a subpopulation. We compute *r_i_*/*n_i_* over all rare SNPs in a gene. We use a linear model with covariates Age and Smoking for trait Q1 and no covariates for Q2, as specified in the simulation model. To avoid sparse data when stratifying by ethnicity, we use a logistic model with no covariates for disease status.

### Scenario 1: common tag SNP, common causal SNP

In the first scenario, the tag SNP detected in the stage 1 GWAS is common and the causal SNP found in the stage 2 sequencing study is also common. We construct a two-stage study combining the GAW17 data set with the HapMap data set as follows. For the stage 1 GWAS, we obtain genotypes at tag SNPs for the individuals in the GAW17 data set (matched by individual ID) from the HapMap data set (build 36, available at http://hapmap.ncbi.nlm.nih.gov) [7]. For the stage 2 sequencing study, we use the GAW17 mini-exome data set for unrelated individuals. We included all 616 subjects for which there was genotype data in both the HapMap build 36 and GAW17 data sets.

For each stage 1 tag SNP, we test for additive genetic effect with trait Q2 using a linear model and select all data sets that are significant at a *p*-value threshold of 0.05. For each data set in which the tag SNP is significant, we estimate the effect at the tag SNP. In stage 2, we estimate the additive genetic effect size for the common causal SNP or rare SNP collapsing statistic *r_i_*/*n_i_* for all data sets in which the tag SNP is significant. We compare the estimates obtained from data sets in which the tag SNP is significant with the estimates obtained for all 200 data sets. PLINK was used for all analyses [8,9].

**Figure 1:**
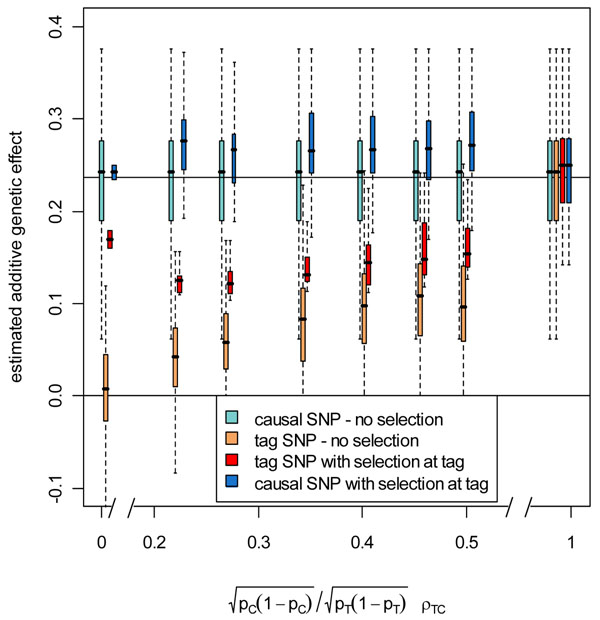
**Distribution of estimates for a common tag SNP and a common causal SNP.** Boxplot of estimates of genetic effect at a tag SNP (GWAS stage 1) and causal SNP C6S5380 (sequencing stage 2) on quantitative trait Q2 over 200 replicates with and without selection at a stage 1 tag SNP for additive genetic effect using a selection threshold *p* < 0.05. *p_C_* is the MAF of the causal SNP, *p_T_* is the MAF of the tag SNP, *ρ_TC_* is the correlation between the tag SNP and the causal SNP. Horizontal lines are the null effect size (zero) and the mean of causal SNP genetic effect estimates without selection. Because of sampling variation, the mean is different from the median (band in middle of boxplots).

We describe the distribution of estimates using a boxplot (Figure 1). We present results for quantitative trait Q2 with causal SNP C6S5380 and tag SNPs from the HapMap data set that fall within the *VNN1* gene, as defined by the gene information file provided with the GAW17 data set. In Figure 1, we present boxplots for the distribution of the genetic effect estimates over 200 replications for six HapMap tag SNPs that are correlated with the causal SNP and one tag SNP that is not correlated with the causal SNP. Because we observed large effect sizes for this uncorrelated tag SNP on both sides of the null value, we used a one-sided test (*α* = 0.025) to ensure that the boxplot would reflect the magnitude of the effect. Using the causal SNP as its own tag, we also include the case in which the tag SNP is perfectly correlated with the causal SNP. In Figure 1 we present 4 boxplots for each tag SNP: (1) the causal SNP genetic effect estimates over all 200 replicates (light blue), (2) the tag SNP estimates over all 200 replicates (orange), (3) the tag SNP estimates for all replicates in which the tag SNP is significant (red), and (4) the causal SNP estimates for all replicates in which the tag SNP is significant (blue).

### Scenario 2: common tag SNP, multiple rare causal SNPs

In the second scenario, the tag SNP is common and there are multiple rare causal SNPs in the same gene at the sequencing stage. We construct a two-stage study from the GAW17 data set using all 697 individuals: The common SNPs are used as tag SNPs and the rare SNPs are used as sequencing SNPs. For each stage 1 tag SNP, we test for additive genetic effect with traits Q1, Q2, and disease status and select all data sets for which the tag SNP *p*-value is smaller than the significance threshold (the threshold was chosen so that estimated power was less than 20%) indicated in Table 1. We estimate the additive genetic effects of the parameter *β* at the tag SNP and the parameter *λ* for the *r_i_*/*n_i_* rare SNP collapsing statistic.

**Table 1:** Bias in genetic effect estimates for a common tag SNP and multiple rare causal SNPs

Trait	Gene	Population	Tag SNP						Rare SNP collapsing statistic *r_i_*/*n_i_*			
		
			SNP	Significance level for additive test	Estimated power for additive test	Mean effect estimate			Correlation between tag SNP and *r_i_*/*n_i_*	Mean effect estimate		
			
						Over all data sets	Over data sets with significant tag effect	Relative bias (%)		Over all data sets	Over data sets with significant tag effect	Relative bias (%)
Q1	*KDR*	CEPH	C4S1878	0.001	0.15	0.63	0.93	49	0.41	5.51	6.19	12
DS	*PIK3C2B*	Tuscan	C1S9170	0.05	0.10	0.87	2.26	160	0.40	6.69	7.08	6
DS	*PIK3C2B*	Tuscan	C1S9171	0.05	0.10	0.87	2.26	160	0.40	6.69	7.08	6
DS	*PTK2B*	CEPH	C8S911	0.05	0.10	0.67	1.49	122	0.51	1.99	2.91	46
DS	*PTK2B*	Chinese	C8S925	0.05	0.06	0.21	1.23	476	0.47	0.34	1.53	350

Results for scenario 2. Values computed as described in the Results section. DS is disease status.

### Scenario 3: tag SNP with MAF 1–5%, multiple rare causal SNPs

In the third scenario, we consider the case in which the tag SNP has a low MAF and there are multiple rare causal SNPs in the same gene in the sequencing stage. We construct a two-stage study from the GAW17 data set using all 697 individuals: Each SNP in a causal gene with MAF between 1% and 5% is in turn used as the GWAS tag SNP; the rest are used as sequencing SNPs (i.e., the tag SNP is excluded from the collapsing statistic *r_i_*/*n_i_*). For each tag SNP, we test for additive genetic effect with traits Q1, Q2, and disease status. We select data sets and estimate effect sizes as in scenario 2.

## Results

We use boxplots or summary measures to describe the effect of selection on the distribution of estimates at the tag SNP and causal SNPs. For scenario 1, we test at a fixed threshold for significance (Figure 1). For scenarios 2 and 3, we select significance thresholds required for low power. Power is estimated as the proportion of data sets for which the tag SNP test is significant. The mean effect estimate is computed over all 200 data sets and is also computed over all data sets for which the tag SNP additive test is significant. We compute the summary measure relative bias as the difference between the mean estimate over data sets for which the tag SNP is significant and the mean estimate over all 200 data sets, divided by the mean estimate over all 200 data sets (Table 1 and 2).

### Scenario 1

The expected pattern of attenuation resulting from imperfect correlation is evident in the distributions of the genetic effect estimates at the tag SNP and causal SNP. The tag SNP estimates (Figure 1, orange boxplots) increase with the *γ_TC_* quantity from equation 1. Selection for significant genetic effect at the tag SNP induces upward bias into the estimate. As *γ_TC_* increases, the power increases and the effect of selection decreases. The difference between the tag SNP estimates with and without selection is much smaller when *γ_TC_* is larger (Figure 1, orange and red box plots). When *γ_TC_* is 0, as for the null case, only the most extreme data sets are selected, and the bias away from the null value is large. When *γ_TC_* is 1, power is high and the bias is smaller.

Because of correlation between the tag SNP and the causal SNP, selection bias in the tag SNP estimate carries through to the causal SNP estimate. Two factors influence the bias at the causal SNP: (1) the correlation between the tag SNP and the causal SNP and (2) the power to detect the effect at the tag SNP. When *γ_TC_* is small, power is low and so the upward bias resulting from selection at the tag SNP is large. As *γ_TC_* increases, power increases and selection bias decreases. On the other hand, when *γ_TC_* is small, the correlation between the tag SNP and causal SNP estimates is small, and so the effect of selection at the tag SNP, although large, carries through to the causal SNP only to a small degree. As *γ_TC_* increases, the correlation increases and the effect of selection, although small, is carried through to a greater degree. These two effects tend to balance each other out so that the bias is similar over the small range of *γ_TC_* in the tag SNPs that we examined. The exceptions are at the extremes. If the tag SNP and the causal SNP are completely uncorrelated, then none of the selection at the tag SNP carries through to the causal SNP (Figure 1, boxplots at far left). If the tag and causal SNPs are perfectly correlated (in our case the tag SNP is in fact the causal SNP), then power to detect the SNP is so high that selection bias is minimal (Figure 1, boxplots at far right).

### Scenario 2

Rare causal SNPs can be difficult to tag with common SNPs because of the upper bound for correlation between two SNPs with different MAFs. In causal genes with multiple rare SNPs, we found 68 SNPs with MAF > 5%. Of these, there were only five SNPs that tagged the effect of the rare SNPs (correlation with *r_i_*/*n_i_* was *ρ* > 0.4 for power to detect effect above significance level *α* for at least one of *α* = 0.001, 0.01, or 0.05). The correlation was low in all five cases (Table 1). The mean tag SNP genetic effect estimate was inflated by selection. Estimates of the rare SNP parameter were biased but to a lesser degree. When there is imperfect correlation between the tag SNP and causal SNPs, some but not all of the selection bias at the tag SNP is transferred to the causal SNP estimate.

### Scenario 3

Rare causal SNPs can be more easily tagged with low-MAF SNPs, because two SNPs with similar MAFs have a higher upper bound for correlation. In causal genes with multiple rare SNPs, we found 162 SNPs with MAF between 1% and 5%. Of these, 12 tagged the effect of the rare SNPs (as defined earlier). The correlation was much higher for these rare tag SNPs than for the common tag SNPs in scenario 2. Bias in the estimate at the tag SNP was, on average, more severe when power was low (Table 2), and relative selection bias at *r_i_*/*n_i_* was also more severe when power was low, although that bias was usually smaller at the causal SNP than at the tag SNP. When we examined cases for which the tag SNP bias was similar (152–186%), bias for the *r_i_*/*n_i_* estimate tended to be higher when correlation was higher. This demonstrates how with higher correlation, more of the tag SNP bias is transferred to the causal SNP.

**Table 2:** Bias in genetic effect estimates for a rare tag SNP and multiple rare causal SNPs

Trait	Gene	Population	Tag SNP						Rare SNP collapsing statistic *r_i_*/*n_i_*			
		
			SNP	Significance level for additive test	Estimated power for additive test	Mean effect estimate			Correlation between tag SNP and *r_i_*/*ni*	Mean effect estimate		
			
						Over all data sets	Over data sets with significant tag effect	Relative bias (%)		Over all data sets	Over data sets with significant tag effect	Relative bias (%)
Q1	*FLT4*	Chinese	C5S5141	0.01	0.075	0.52	1.00	94	0.46	3.26	4.05	24
Q2	*PLAT*	Luhya	C8S1797	0.05	0.105	0.29	0.98	241	0.46	0.37	1.71	356
Q2	*SREBF1*	CEPH	C17S1023	0.05	0.135	0.59	1.63	177	0.81	2.00	3.97	98
Q2	*VLDLR*	Tuscan	C9S404	0.01	0.055	0.84	2.28	173	0.51	0.99	2.53	155
Q2	*VLDLR*	Tuscan	C9S471	0.01	0.055	0.84	2.28	173	0.51	0.99	2.53	155
Q2	*VWF*	CEPH	C12S193	0.01	0.070	0.34	0.93	171	0.65	0.61	1.12	85
Q2	*VWF*	CEPH	C12S200	0.01	0.080	0.41	1.15	182	0.61	0.57	1.04	84
Q2	*VWF*	CEPH	C12S203	0.05	0.075	0.21	0.53	157	0.48	0.63	0.93	47
Q2	*VWF*	CEPH	C12S211	0.01	0.070	0.34	0.87	152	0.78	0.65	1.39	113
Q2	*VWF*	Japanese	C12S193	0.05	0.070	0.21	0.72	244	0.56	-0.12	0.36	403
Q2	*VWF*	European	C12S193	0.05	0.200	0.31	0.79	153	0.62	0.55	1.03	87
Q2	*VWF*	European	C12S200	0.01	0.070	0.30	0.92	203	0.45	0.49	0.95	93
Q2	*VWF*	European	C12S203	0.05	0.070	0.18	0.50	180	0.46	0.58	0.85	47
Q2	*VWF*	European	C12S211	0.01	0.055	0.32	0.90	186	0.74	0.59	1.22	107
Q2	*VWF*	All	C12S203	0.05	0.140	0.16	0.41	160	0.48	0.69	1.06	54
DS	*PTK2B*	CEPH	C8S883	0.05	0.075	0.95	2.40	153	0.53	2.35	13.83	489
DS	*PTK2B*	Chinese	C8S885	0.05	0.050	0.16	1.24	692	0.87	0.38	2.68	610
DS	*PTK2B*	European	C8S911	0.05	0.130	0.63	1.27	102	0.50	2.04	2.86	40

Results for scenario 3. Values computed as described in the Results section. DS is disease status.

## Discussion

Our mini-exome two-stage study design was different from a genome-wide two-stage study design in several ways. The sample size was small (hundreds instead of thousands of SNPs), the *p*-value threshold for selection was large (*α* = 0.05 instead of 10^−6^), and the correlation was less than ideal in most cases. The low correlation resulting from linkage disequilibrium between the causal and tag SNPs attenuated the effect size at the tag SNP, causing tag SNP estimates to fall well below the true causal SNP effect size. With a better set of tag SNPs, the attenuation would be less severe. To obtain an adequate number of significant data sets for each tag SNP, we used a liberal threshold for selection. A genome-wide significance level (10^−6^) would have decreased power, causing selection bias to be more severe.

Dickson et al. [10] demonstrated how multiple rare causal SNPs can be correlated with a single common tag SNP and can produce an apparent association at that tag SNP. Their simulation studies also showed that this synthetic association can occur over long ranges, often longer than would be covered by targeted resequencing around an associated GWAS SNP. In our study, we searched for tag SNPs that were correlated with *r_i_*/*n_i_* within the same gene and did not find more than two. In practice, expanding the examined regions may detect additional tag SNPs correlated with the *r_i_*/*n_i_* statistic for the region, but at the cost of additional sequencing.

Recently, methods have been proposed to correct for upward bias in genetic effect estimates at a GWAS tag SNP caused by selection at that same tag SNP [3,11]. Extensions of the methods are needed for two-stage designs in which upward bias at one or more stage 2 sequencing SNPs is caused by selection at an imperfectly correlated stage 1 GWAS tag SNP. Selection bias is of practical importance when designing a replication study. If the sample size is estimated from an upward-biased estimate of effect size at the causal SNP, then the replication study may be underpowered to detect the true association. Reliable estimates of genetic effect are also important for clinical interpretation and estimation of the proportion of heritability explained.

## Conclusions

Targeted resequencing following a GWAS is becoming a cost-effective way to uncover causal variants not included in commercial genotyping chips. Because of low power, many association studies will be of low to moderate significance and follow-up studies will be required to confirm the associations. If the causal SNP genetic effect estimate in the original study is biased, then power to detect this SNP in a follow-up study will also be overestimated, and true associations not replicated because of low power may be misinterpreted as null associations. One might expect that when tag and causal SNPs are not well correlated, the effect of selection on the causal SNP estimate will be negligible. Our work indicates that this is not the case. To avoid discarding disease-associated SNPs in the follow-up stage, it is critical that investigators account for selection bias in the original resequencing study.

## Competing interests

The authors declare that there are no competing interests.

## Authors’ contributions

LF and SBB conceived of the study. LF designed and carried out the analyses and drafted the manuscript. All authors read and approved the final manuscript.
